# Editorial: The role of sphingolipid metabolism in endocrine diseases

**DOI:** 10.3389/fendo.2024.1506971

**Published:** 2024-10-30

**Authors:** Cao Li, Guangbi Li

**Affiliations:** ^1^ Department of Pharmacy, Beijing Tiantan Hospital, Capital Medical University, Beijing, China; ^2^ Department of Pharmacology and Toxicology, School of Medicine, Virginia Commonwealth University, Richmond, VA, United States

**Keywords:** sphingolipid, diabetes, obesity, glucose homeostasis, lipidomics

In 1874, Johann Thudichum described sphingolipids as unusual “Sphinx-like” lipids for its enigmatic properties (1). Decades ago, sphingolipids are recognized as essential components of cell membranes maintaining the structural stability of cell and organelle membranes. In recent years, variety of sphingolipids were known playing critical role in regulating key processes of pathophysiological functions. Today, the balance between different sphingolipid species is known crucial for modulating the human health and disease. Recent advances in multi-omics-based analyses and methodologies have further revealed that endocrine system and energy metabolism are closely linked to sphingolipid homeostasis, and disease-specific alterations in sphingolipids and their associated enzymes can serve as prognostic markers for the progression of human diseases (2).

Classic endocrine diseases typically include diabetes, hyperthyroidism etc. However, with the evolving understanding of disease mechanisms, the definition of endocrine diseases has expanded to include fertility-related endocrinology, immune-endocrinology, neuroendocrinology. This broadened definition has incorporated a range of metabolic, immune, neural, and environmentally-related diseases under the umbrella of endocrine disorders. Human clinical data from genome-wide association studies and preclinical data from disease models suggest that sphingolipids may be a key factor in enhancing our understanding of endocrine diseases.

Population and aging issues are significant social and economic concerns worldwide, especially in certain East Asian and European countries (3). With the acceleration of modern life and increased work-related stress, male infertility has become a prominent public health issue. Wang et al. comprehensively reviews recent studies on the crosstalk between sphingolipid metabolites and steroid hormone homeostasis. The authors provide an in-depth analysis of the role of various sphingolipid metabolites, such as ceramide, sphingosine, sphingosine-1-phosphate (S1P), and sphingomyelin, in regulating sexual steroidogenesis and their impact on the development and pathology of male infertility. The paper elucidated multiple biochemical pathways involving sphingolipid metabolites, such as the cAMP/PKA signaling pathway, cytokine-mediated signaling pathways (e.g., TNF-α, IL-1β), and the PI3K/ERK pathway, demonstrating the complexity of sphingolipid involvement in steroid hormone synthesis and deepening the understanding of how these pathways intersect and influence each other. In addition, various sphingolipids metabolites participate in different stages of spermatogenesis, especially ceramide and S1P, in mediating testicular cell apoptosis and testis injury.

Graves’ disease is an autoimmune disorder characterized by excessive thyroid hormone secretion, which often impairs in the eyes. Gulbins et al. present a compelling analysis of the complex role of sphingolipids in the pathophysiology of thyroid eye disease (TED) and other autoimmune diseases such as Graves’ disease (GD). The authors systematically described the contribution of various sphingolipid metabolites, such as ceramide, sphingosine, and S1P, to the pathological mechanisms driving TED. They highlight the dual roles of ceramide and S1P in mediating inflammation and fibrosis in the orbital tissues of patients with TED. The article effectively links sphingolipid dysregulation to the clinical symptoms of TED, such as orbital inflammation. Mechanistically, the authors explore the role of acid or neutral sphingomyelinase in autoimmune diseases, how it promotes the function of regulatory T cells (Tregs), CD4^+^, and CD8^+^ T cells, and consequently modulates the immune response. Further, Gulbins et al. examined the therapeutic potential of linsitinib in the treatment of TED. Using experimental mouse models, the study elucidates the effects of linsitinib, a small molecule kinase inhibitor of the insulin-like growth factor 1 receptor (IGF-1R) and insulin receptor (IR), on disease progression, autoimmune responses, and tissue remodeling. Previous studies from the same group have demonstrated that sphingolipids, particularly sphingosine 1-phosphate, play a crucial role in orbital tissue remodeling following TED induction (4). It would be interesting to investigate whether linsitinib also influences this pathway and modulates the orbital sphingolipids.

Sphingolipids are closely linked to glucose metabolism and insulin resistance, especially brain insulin resistance (BIR). Mei et al. have connected dysregulated sphingolipid metabolism to the development and progression of BIR, which is associated with various neurological diseases such as Alzheimer’s disease (AD) and Parkinson’s disease (PD). The authors first described the complex roles played by different types of sphingolipids in various cell types within the central nervous system, including neurons, microglia, and astrocytes. Phenotypically, dysregulated sphingolipid metabolism is associated with amyloid-β (Aβ) plaques, tau hyperphosphorylation, and α-synuclein aggregation. From a mechanistic perspective, the authors effectively discuss how disruptions in sphingolipid pathways, especially those involving ceramide, S1P, glucosylceramide, and sphingomyelin, lead to the development of BIR and subsequently increase the risk of neurodegenerative diseases such as AD and PD.

Metabolic dysfunction-associated fatty liver disease (MASLD) is a significant cause of chronic liver disease, and is closely associated with obesity and related metabolic disorders. Although the FDA approved new drugs for the treatment of the progressive subtype of MASLD (metabolic dysfunction-associated steatohepatitis, MASH) in 2024, the approval was conditional (5). Ramos-Molina et al. discussed the role of sphingolipids as key drivers of MASH and their involvement in disease mechanisms. Under pathological conditions, the accumulation of ceramide in hepatocytes triggers inflammatory pathways, disrupts insulin signaling, and promotes liver fibrosis through apoptosis and autophagy. Notably, this review thoroughly examines the role of traditional and emerging potential therapeutic medications for MASLD, including drugs targeting sphingolipid metabolism, such as Myriocin, Fingolimod, and Fumonisin B1, as well as glucose-lowering agents like Metformin, GLP-1 receptor agonists, and SGLT-2 inhibitors. The authors also address the limitations and challenges in translating these findings into clinical practice, providing an important reference for clinicians and researchers.

In conclusion, sphingolipids research provides a novel perspective for understanding the pathological mechanisms of complex endocrine and related diseases and lays the groundwork for developing new diagnostic and therapeutic strategies. Additionally, optimizing therapeutic strategies targeting sphingolipid metabolism and integrating emerging multi-omics technologies and biomarker research will help advance clinical translation in this field.
